# Ferroelectric and Optoelectronic Coupling Effects in Layered Ferroelectric Semiconductor‐Based FETs for Visual Simulation

**DOI:** 10.1002/advs.202413808

**Published:** 2025-01-22

**Authors:** Can Zhao, Zhaotan Gao, Zian Hong, Hongzhi Guo, Zhili Cheng, Yawei Li, Liyan Shang, Liangqing Zhu, Jinzhong Zhang, Zhigao Hu

**Affiliations:** ^1^ Technical Center for Multifunctional Magneto‐Optical Spectroscopy (Shanghai), Engineering Research Center of Nanophotonics & Advanced Instrument (Ministry of Education), Department of Physics, School of Physics and Electronic Science East China Normal University Shanghai 200241 China

**Keywords:** 2D ferroelectric semiconductor, 2H α‐In_2_Se_3_, in‐memory sensing and computing, memory retention, optoelectronic coupling effects

## Abstract

Controlling polarization states of ferroelectrics can enrich optoelectronic properties and functions, offering a new avenue for designing advanced electronic and optoelectronic devices. Here, ferroelectric semiconductor‐based field‐effect transistors (FeSFETs) are fabricated, where the channel is a ferroelectric semiconductor (e.g., α‐In_2_Se_3_). Multiple conductance states are achieved in α‐In_2_Se_3_‐based FeSFETs by controlling the ferroelectric polarization. The on/off current ratio (I_on_/I_off_) is ≈10^5^ with a dark current of ≈10^−11^ A by applying a single positive gate voltage pulse. Moreover, the device shows excellent endurance and retention performance. In a further step, the carrier transports and corresponding physics mechanism in various polarization states are studied by using Kelvin probe force microscopy (KPFM) and optoelectronic measurements. Finally, the α‐In_2_Se_3_‐based FETs can be trained. It can recognize handwritten digit images from MNIST dataset with a successful recognition accuracy of ≈95.5%. This work provides a new design idea and theoretical support for advanced optoelectronic devices in the field of in‐memory sensing and computing.

## Graphical abstract

A α‐In_2_Se_3_‐based FeSFET is developed to realize optical storage in various polarization states. The unique ferroelectricity has an excellent electric performance (Ion/Ioff = 105) and device stability. Moreover, the ferroelectric and optoelectronic coupling effects in FeSFETs are clarified to simulate the persistent visual behavior in the nervous system with a high recognition accuracy of ≈95.5%.

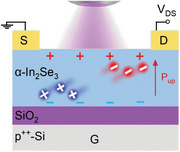

## 1 Introduction

Ferroelectrics, such as Pb(Zr_*x*_Ti_1 − *x*_)O_3_, BiFeO_3_, (Hf_*x*_Zr_1 − *x*_)O_2_, and polyvinylidene fluoride (PVDF), exhibit spontaneous electric polarization, which can be tuned by applying an external electric field.^[^ ^1^, ^2^, ^3^, ^4^, ^5^ ^]^ They are extensively used in storage devices, actuators, sensors and energy storage,^[^ ^6^, ^7^, ^8^ ^]^ offering a wide range of physical and technological applications in nanoelectronics.^[^ ^9^, ^10^, ^11^ ^]^ For the case of information storage, ferroelectrics are in capacitor and/or field effect transistor (FETs) configurations.^[^ ^12^, ^13^ ^]^ However, the depolarization field significantly affects ferroelectricity when the ferroelectric thickness is reduced to a few nanometers. Moreover, conventional ferroelectric FETs consist of a ferroelectric insulator cooperating with a semiconductor as the channel material. This combination suffers from the short retention time, which limits the practical applications in memory and storage.^[^ ^14^ ^]^

Recently, 2D van der Waals (vdWs) layered ferroelectrics such as CuInP_2_S_6_, GeS, SnS, and SnSe have attracted significant attention due to their stable ferroelectric properties even in atomically layers.^[^ ^15^, ^16^, ^17^, ^18^, ^19^, ^20^, ^21^, ^22^, ^23^ ^]^ Specifically, the clean vdW interfaces and narrow bandgap provide a distinct advantage compared to conventional ferroelectrics.^[^ ^14^ ^]^ The vdW interfaces enable integration with other 2D materials, resulting in innovative heterostructures with special electronic properties.^[^ ^24^ ^]^ Meanwhile, narrow bandgaps are essential for low‐power electronic and optoelectronic applications, facilitating efficient switching and modulation.^[^ ^25^ ^]^

Among vdW ferroelectric semiconductors, α‐In_2_Se_3_ stands out as a promising candidate for the channel materials of FETs owing to its multidirectional stable ferroelectricity. Xue et al. observed electric‐field induced polarization switching and hysteresis loop in double layer and single layer α‐In_2_Se_3_, respectively.^[^ ^26^ ^]^ The ferroelectricity of α‐In_2_Se_3_ arises from the relative displacement of the central Se atomic layer with respect to the adjacent in atomic layers.^[^ ^27^, ^28^, ^29^, ^30^, ^31^ ^]^ Moreover, the strong photoresponse and highly tunable electronic bandgap become advantageous from an optoelectronics point‐of‐view,^[^ ^32^, ^33^ ^]^ extensively used in optoelectronic memories. Although employing the floating‐gate structure is an effective method to realize the memory function in optoelectronic transistors, the three‐terminal structure limits the integration density and requires large programming voltage.^[^ ^34^ ^]^ Importantly, the memory function of these devices relies on charge trapping of photoexcited carriers in defects and impurities on surface or/and interface, which results in short retention times and sensitivity to environmental factors. Compared to conventional semiconductors,^[^ ^35^, ^36^, ^37^ ^]^ α‐In_2_Se_3_ ferroelectric semiconductor has attracted much attention for its unique photoelectric coupling properties. The ferroelectric storage performance has been demonstrated by electrical and optical pulse methods based on the optical properties of α‐In_2_Se_3_‐based FETs.^[^ ^38^, ^39^ ^]^ The coexistence of bound and mobile charges in α‐In_2_Se_3_ makes it possible to change the polarization state of the materials by incident light, providing a rich physical connotation for photoelectric storage.^[^ ^40^, ^41^ ^]^

In this study, the FETs with a single ferroelectric semiconductor as the channel (FeSFET) were fabricated to realize optical storage in various polarization states. The device demonstrates remarkable non‐volatile memory properties, including robust long‐term retention (>5000 s), superior endurance (>2000 cycles). In addition, the internal optoelectronic coupling mechanism of α‐In_2_Se_3_‐based FeSFETs have been investigated to simulate the persistent visual behavior in the nervous system. By taking advantage of dynamic response to light signals, the α‐In_2_Se_3_‐based FETs can train and recognize handwritten digit images from MNIST dataset with a high recognition accuracy of ≈95.5%. This work provides a new design idea for the discovery of non‐volatile high‐density memory and multi‐field sensing memory, which lays a foundation for in‐memory sensing and computing.

## 2 Results and Discussion

### 2.1 Electric Performance of FeSFETs

**Figure 1:**
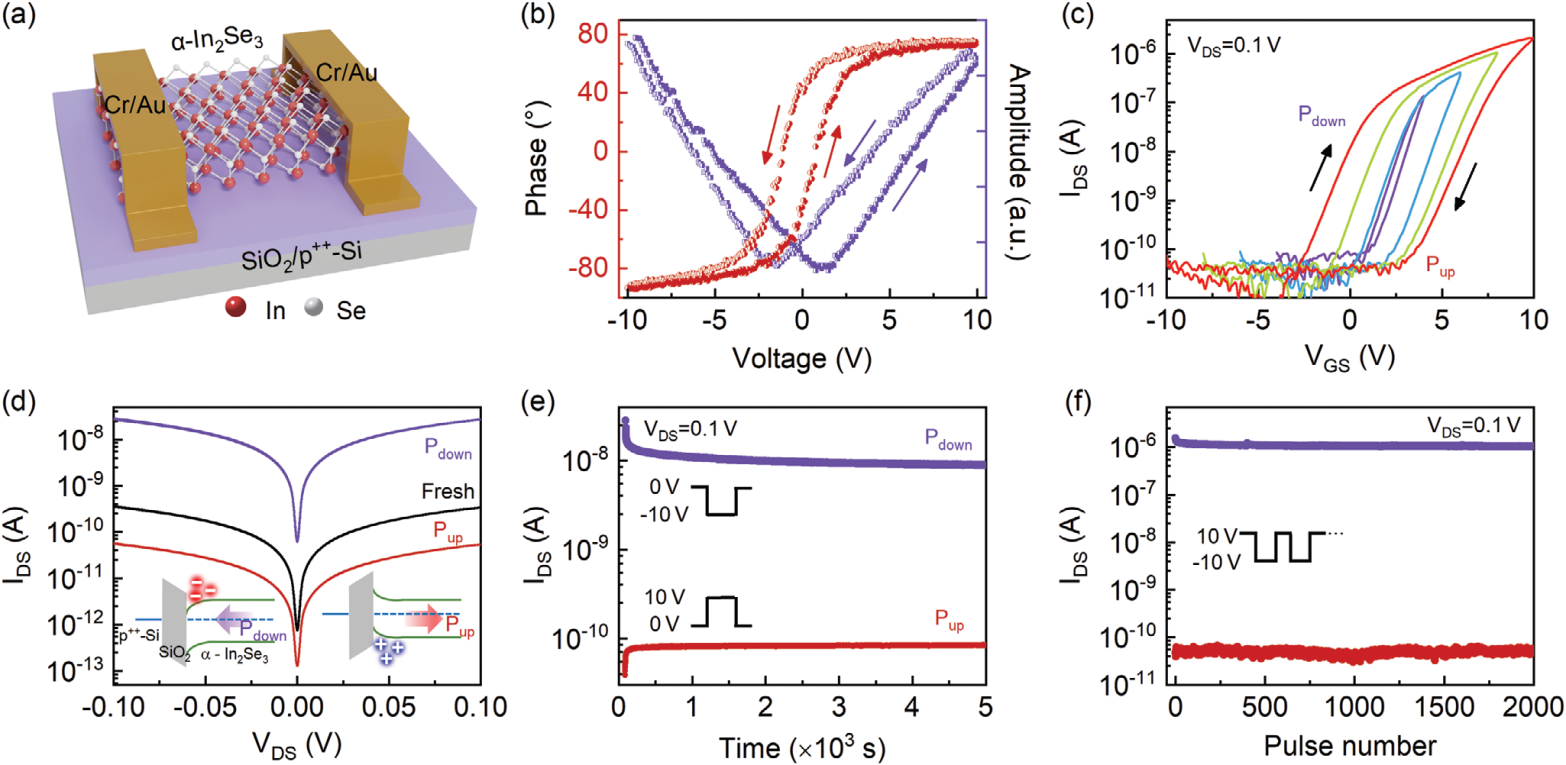
Structure and electric performances of an α‐In_2_Se_3_‐based FeSFET. a) Schematic diagram of a FeSFET. b) PFM phase and amplitude of a 2H α‐In_2_Se_3_ nanosheet. c) Transfer characteristic curves of a FeSFET at V_DS_ = 0.1 V. d) Forward and backward swept output characteristic curves (V_GS_ = 0) in three polarization states: fresh state (unpolarization), polarization up (P_up_), and polarization down (P_down_). Energy band diagrams for the cases of P_up_ and P_down_. e) Time‐dependent I_DS_ recorded at V_GS_ = 0 V after a single V_GS_ pulse (V_GS‐preset_ = ±10 V, pulse width: 5 s), f) Cycle‐to‐cycle stability tested at V_GS‐preset_ = 10 V or –10 V by applying V_GS_ pulses (10 V to –10 V to 10 V, pulse width: 300 ms).

**Figure** **1**a and Figure S1a (Supporting Information) show a schematic diagram and an optical microscopic image of a FeSFET (Au/α‐In_2_Se_3_/SiO_2_/p^++^‐Si) with the ferroelectric semiconductor α‐In_2_Se_3_ as a channel, respectively. Atomic force microscopy (AFM) images and the corresponding line profiles (Figure S1b, Supporting Information) indicate that the thickness of α‐In_2_Se_3_ nanoflakes is ≈26 nm. The four first‐order Raman peaks in Figure S1c (Supporting Information) nearby 90, 106, 187, and 194 cm^−1^ come from the E_2_, A_1_(LO+TO), A_1_(LO) and A_1_(TO) phonon modes of α‐In_2_Se_3_ with a hexagonal structure (2H), respectively.^[^ ^42^, ^43^, ^44^ ^]^ Additionally, the phase and amplitude hysteresis loops of piezoelectric force microscopy (PFM) show that α‐In_2_Se_3_ exhibits excellent ferroelectric properties, as shown in Figure 1b. The transfer characteristic curves reveal an *n*‐type feature of the α‐In_2_Se_3_‐based transistor (cf. Figure 1c). It suggests that the polarization is precisely controlled since the hysteresis loops (window width and current amplitude) become large with increasing the sweeping range of V_GS_. The α‐In_2_Se_3_‐based FeSFET exhibits an impressive on/off current ratio (I_on_/I_off_ ∼ 10^5^). The polarization up (P_up_) and polarization down (P_down_) states are achieved by applying gate voltage pulses (+10 V and –10 V), respectively. In Figure 1d, forward and backward swept output characteristic curves are affected by the different polarization states. It reveals that the device exhibits a lower output current in the P_up_ state than those in the other two states. The corresponding physics mechanism under both polarization states (P_up_ and P_down_) is shown in the inset of Figure 1d and Figure S2 (Supporting Information). For the bottom‐gate configuration, an upward (downward) polarization of α‐In_2_Se_3_ is induced near its interface with SiO_2_ when a high positive (negative) gate voltage is applied. Therefore, a sheet of negative (positive)‐bonded charges at the bottom surface of α‐In_2_Se_3_ is produced, resulting in an energy band bending upward (downward).^[^ ^42^ ^]^ Band bending causes charge depletion (accumulation) in α‐In_2_Se_3_, which results in a lower (higher) current level. Note that the channel current of the α‐In_2_Se_3_‐based ferroelectric transistor is independent of the ferroelectric polarization order (Figure S3, Supporting Information). It means that the application order of the pre‐gate voltage pulse does not affect the device performance, which is confirmed by the cycle‐to‐cycle stability tests (Figure 1f). Meanwhile, the in‐plane ferroelectric polarization can be neglected due to the overlap of output curves swept forward and then backward (–0.1 V → 0.1 V → –0.1 V).

The post‐pulse current in the two polarization states (P_up_: V_GS_ = 10 V, P_down_: V_GS_ = –10 V; pulse width: 5 s) are investigated for the FeSFET electrical storage capability. Figure 1e shows that the dark current was suppressed and reduced to 10^−11^ A due to the remnant polarization (P_up_) of α‐In_2_Se_3_. On the other hand, the current level remains above 10^−8^ A after 5000 s in the P_down_ state. Note that the on/off current ratio between the P_down_ and P_up_ states increases as the amplitude of gate voltage pulses increases. The ratio between the two states exceeds 10^2^, indicating a large dynamic range (cf. Figure S4, Supporting Information). In Figure 1f, the device exhibits excellent cycling stability, maintaining a switching ratio of ≈10^4^ for 2000 consecutive cycles. It indicates that the α‐In_2_Se_3_‐based FeSFETs are potential candidates for high‐performance computing and memory devices.

### 2.2 Photoresponse and Retention in P_up_ and P_down_ States

**Figure 2:**
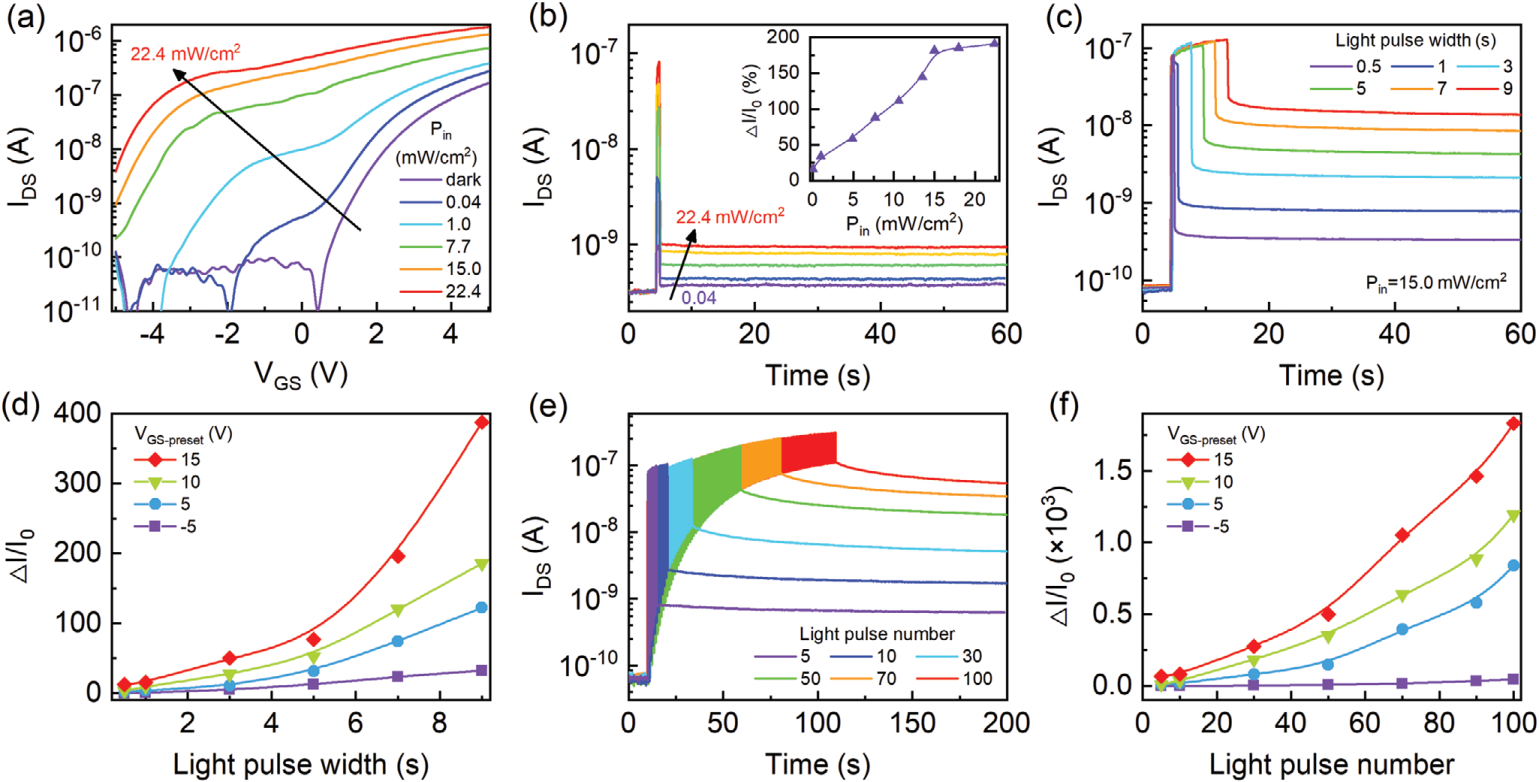
Optoelectronic performance of an α‐In_2_Se_3_‐based FeSFET. a) Transfer characteristic curves of the device illuminated by various light power densities (λ = 405 nm). b) Time‐dependent I_DS_ by applying a single light pulse with different P_in_ from 0.04 to 22.4 mW cm^−2^ (pulse width: 0.5 s). Inset: ΔI/I_0_ as a function of light power density. c) Time‐dependent I_DS_ by applying a single light pulses with different light pulse widths (P_in_ = 15.0 mW cm^−2^) after a V_GS_ pulse (V_GS‐preset_ = 10 V, pulse width: 3 s) and d) the corresponding ΔI/I_0_ as a function of light pulse width. e) Time‐dependent I_DS_ by applying multi‐pulsed light with different pulse number and f) the corresponding ΔI/I_0_ as a function of light pulse number.

**Figure** **2**a shows the transfer characteristic curves at different laser power densities to study the effect of gate voltage on the photoresponse of α‐In_2_Se_3_‐based FeSFET. Under dark conditions, the channel current increases with increasing the positive gate voltage with a cut‐off characteristic at negative gate voltage. It indicates that α‐In_2_Se_3_ has an n‐type characteristic. Under illumination, the current level increases gradually. The cut‐off voltage has a shift towards to negative gate voltage under light illumination since α‐In_2_Se_3_ produces photogenerated carriers. Note that the cut‐off voltage increases from 0.2 V under dark conditions to –1.6 V at P_in_ = 0.04 mW cm^−2^. The α‐In_2_Se_3_‐based FeSFET has different photocurrent gain under positive and negative gate voltages due to the difference out‐of‐plane polarization in α‐In_2_Se_3_ (Figure S5, Supporting Information). Figure 2b reveals that the I_DS_ current increases as the laser pulse width increases. After illumination, I_DS_ does not return to the initial level, which indicates that the α‐In_2_Se_3_‐based FeSFET exhibits optical storage capability. The retention capability was evaluated after the application of various pulsed gate voltages. The retention capability index is calculated according to the formula: ΔI/I_0_ = (I‐I_0_)/I_0_ ×100%, where I and I_0_ represent the current amplitudes after and before illumination, respectively. In the inset of Figure 2b, the retention capability increases and tends to be saturated at the P_in_ above 15 mW cm^−2^. Therefore, the light power density P_in_ = 15 mW cm^−2^ is fixed in the following optoelectronic measurements.

In a further step, polarization‐dependent optoelectronic performance of the α‐In_2_Se_3_ based FeSFETs have been investigated in various ferroelectric polarization states. Figure 2c shows the optoelctronic performance of a FeSFET at V_DS_ = 0.1 V and various laser pulse width. As the pulse width increases from 0.5 to 9 s, the cumulative effect becomes more pronounced, leading to a higher ΔI/I_0_. Note that I_0_ is the I_DS_ before illumination and ΔI is the difference of I_DS_ before and after illuminations (cf. Figures S6, Supporting Information). In Figure 2d, ΔI/I_0_ increases with the laser pulse widths and gate voltage spikes. Especially, it increases from 30 to 500 as the voltage increase from –5 to 15 V when the single laser pulse width is 10 s. Furthermore, the memory retention capability can be enhanced when triggered with repeated light pulses. The current responses and relaxation time curves for different light pulse numbers are shown in Figure 2e. As the pulse number increases from 5 to 100, the accumulation of photo‐generated carriers do not dissipate quickly, which hinders I_DS_ from returning its initial state (I_0_), resulting in an increase of the ΔI/I_0_ index. For the case of multiple pulses, it increases from 44 to 1.8× 10^3^ as the gate voltage increase from –5 to 15 V when the light pulse number is 100, as shown in Figure 2f. It indicates that ΔI/I_0_ in the P_up_ state is significantly higher than that in the P_down_ state. Therefore, the FeSFET exhibits super memory retention capacity in the P_up_ state. Note that ferroelectric polarization in α‐In_2_Se_3_ is preset to the P_up_ state by applying a pulsed gate voltage of 10 V (pulse width: 3 s) for the optoelectronic measurements. Figure S6a presents the output characteristic curves (I) before and (III) after illumination (P_in_ = 15 mW cm^−^2) under the two ferroelectric polarizations (P_down_ and P_up_). In the P_down_ state, ΔI = 10.8 nA is obtained at V_DS_ = 0.1 V, resulting in a low ΔI/I_0_ ratio (<1) due to a large dark current. On the contrary, the dark current in the P_up_ state is suppressed, leading to a much higher ΔI/I_0_ ratio of ≈20. The charge retention capability of the device increases with increasing the gate voltage pulse width (Figure S7, Supporting Information). The cycle‐to‐cycle and cell‐to‐cell tests show that the α‐In_2_Se_3_‐based FeSFETs could maintain the photoelectric performance after multiple reset operations and a good repeatability (Figure S8, Supporting Information).

### 2.3 Optoelectronic Coupling Effects in the FeSFETs

**Figure 3:**
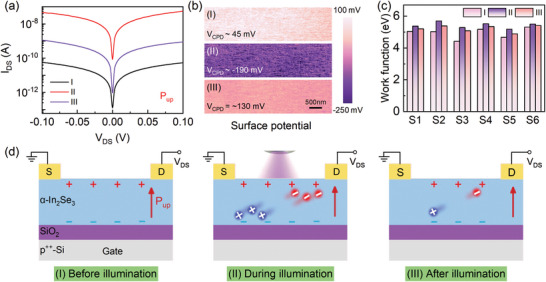
Output characteristic curves and the corresponding physic mechanism of α‐In_2_Se_3_‐based FeSFETs under various light conditions. a) Output characteristic curves of a FeSFET I) before II) during and III) after illumination in a P_up_ state. b) KPFM surface potential images with the scan size 5 µm × 1.5 µm and c) the corresponding work function of 2H α‐In_2_Se_3_ under the three illumination conditions. d) The distributions of ferroelectric polarization‐ and photon‐induced carriers under the three light conditions in a P_up_ state.

**Figure** **3**a shows the output characteristic curves of α‐In_2_Se_3_‐based FeSFETs in the P_up_ state and three light conditions: (I) before illumination, (II) during illumination with a 405 nm‐laser, and (III) after illumination. The I_DS_ current level is improved by more than three orders of magnitude under illumination. Kelvin probe force microscopy (KPFM) is an effective technique for obtaining topographic and localized contact potential difference (CPD) between the tip and sample surface. Note that the KPFM scanning region is a part of the FET channel (i.e., α‐In_2_Se_3_). The CPD was measured by applying a voltage to the tip. The sample work function was derived from the formula^[^ ^45^ ^]^: V_CPD_ = (ϕ_tip_ − ϕ_sample_) / *e*, where *e* is the elementary charge, ϕ_tip_ and ϕ_sample_ are the work functions of the tip and sample, respectively. Note that the device was set to a P_up_ state by applying a V_GS_ = 10 V pulse. In order to investigate the illumination‐dependent surface potential of α‐In_2_Se_3_, the CPD under three conditions were calculated, as shown in Figure 3b. The average V_CPD_ value of the channel changes from 45 mV under dark condition, to –190 mV during illumination, and finally to –130 mV after illumination due to the combination of ferroelectric polarization‐ and photon‐induced carries. Note that V_CPD_ value (–130 meV) under the III condition remains lower than that (45 meV) under the I condition. Moreover, the variation of surface potential for α‐In_2_Se_3_ devices is mainly due to the change of illumination conditions instead of ferroelectric depolarization (Figure S9, Supporting Information). In Figure 3c, the work function of α‐In_2_Se_3_ increases with the addition of illumination and decreases after illumination, while it still remains higher than the initial state, which is confirmed by different devices. The phenomenon is consistent with the V_CPD_ results.

Figure 3d depicts the distributions of ferroelectric polarization‐ and photon‐induced charges in the ferroelectric semiconductor channel under different light conditions. In the P_up_ state before illumination as shown in Figure 3d(I), the positive polarization‐induced bound charges accumulated on the top surface of α‐In_2_Se_3_ which results in a relatively positive surface potential. The ferroelectric polarization in the ferroelectric channel has a built‐in electric field (E_in_). During the illumination, the photon‐induced excitons shown in Figure 3d(II) will be separated and transported by the E_in_. It means the photon‐induced electrons will move to the channel surface and the photon‐induced wholes move to the channel bottom, which will significantly reduce the P_up_ field. The reduce P_up_ state will be restored once the light is removed (Figure 3d III). For the case of P_down_ state, the negative bound charges are accumulated on the top surface of α‐In_2_Se_3_, which results in a relatively negative surface potential before illumination (Figure S10a, Supporting Information). In Figure S10b (Supporting Information), the E_in_ generated by ferroelectric polarization plays an important role in separating and transporting photon‐induced excitons. The semiconductor‐dielectric interface will accumulate electrons, while positive bound charges on the top surface of α‐In_2_Se_3_ result in a positive shift in surface potential. Note that the non‐volatile photoconductivity is mainly caused by ferroelectric polarization and photo‐induced charges instead of defect states at the interface between In_2_Se_3_ and SiO_2_, which is confirmed by the In_2_Se_3_/*h*‐BN/SiO_2_ device structure (cf. Figure S11, Supporting Information). Therefore, illumination and electronic polarization field could manipulate the carriers in the α‐In_2_Se_3_ channel of FeSFETs. The coupling mechanism of ferroelectric and photoelectric has been confirmed by the PFM and photocurrent mapping (cf. Figures S12 and S13, Supporting Information).

### 2.4 Simulation of the Vision Persistence Behavior

**Figure 4:**
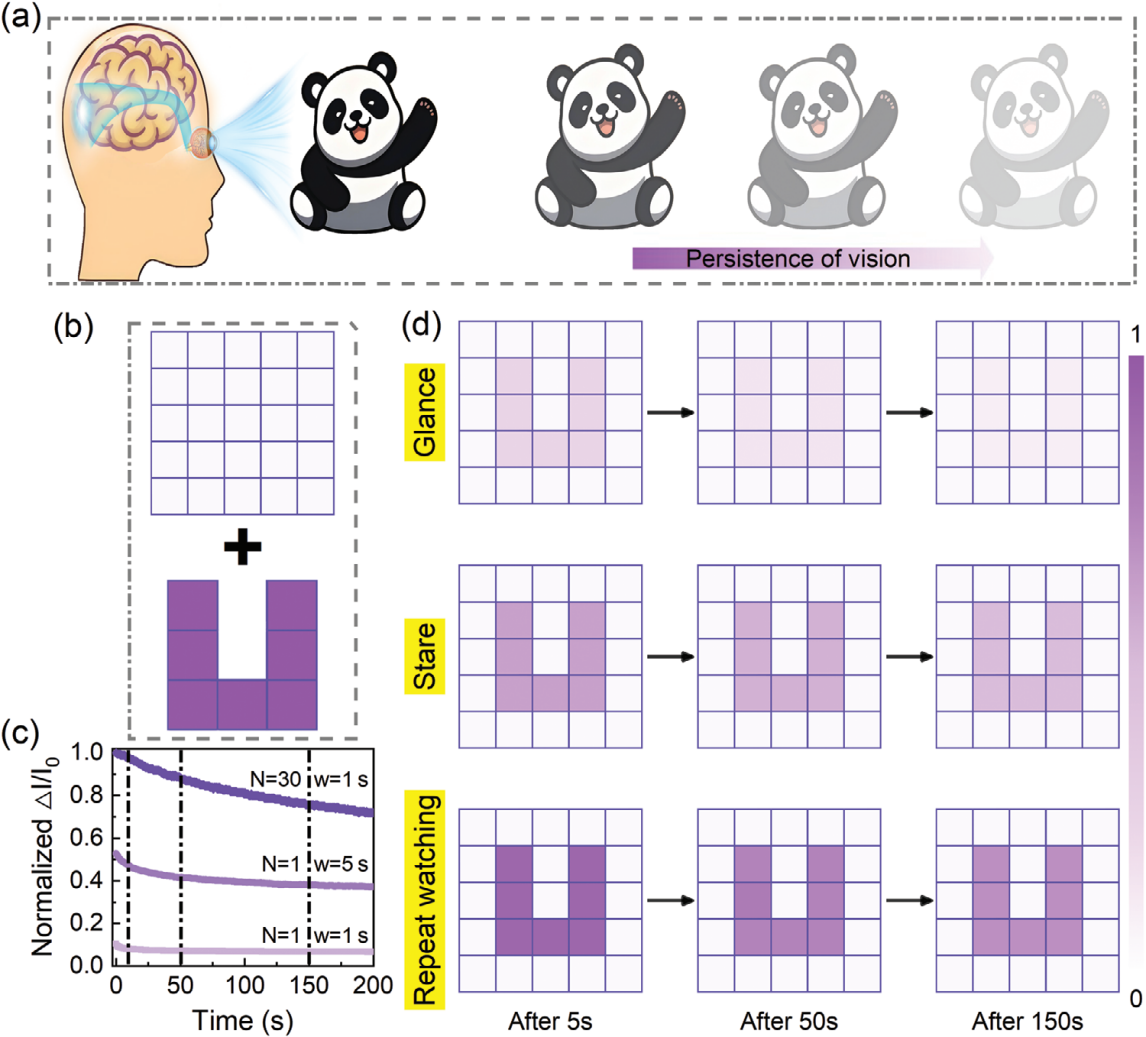
Stimulation of the vision persistence behavior of FeSFETs. a) Schematics diagram of visual persistence of human eyes. b) The background and input signal``U″. c) Normalized ΔI/I_0_ after pulsed laser with different pulse width and number (λ = 405 nm, P_in_ = 15 mW cm^−2^, V_DS_ = 0.1 V). d) The visual persistence after moving illumination (405 nm, 15 mW cm^−2^) with different pulse width/number for 5, 50, and 150 s.

In **Figure** **4**a, visual images are formed through the short‐term storage and brain′s processing of visual stimuli information, involving the conversion of external light signals into neural signals via retina, and then transmitted to brain for processing.^[^ ^46^, ^47^, ^48^ ^]^ Visual persistence refers to the visual sensory memory phenomenon where a stimulus appears to remain visible after it has been removed.^[^ ^49^ ^]^ Similar to human visual persistence, the retention capability triggered by a light stimulus also exhibits a slow decay when the stimuli are removed, which can be used to simulate visual persistence in the neural system.^[^ ^50^ ^]^ Visual persistence properties during vision formation successfully are emulates based on the α‐In_2_Se_3_‐based FeSFETs, which is illuminated by a 405 nm‐laser pulse in the P_up_ state. In Figure 4b, the character “U” is used as the input signal, and an image of this character is immediately formed after stimulation. The imaging evolution process was investigated with an increase in illumination time, light pulse number, and the time after illumination (Figure 4c; Figure S14, Supporting Information). It suggests that the normalized ΔI/I_0_ increases as the pulse width and number increase and decays slowly after illumination. Figure 4d displays a discernible current variation when the device is presented with a fleeting impression of the letter “U,” analogous to a short period of illumination (< 1 s) to mimic glance. Even after the visual signal for 150 s, the “U” shape is retained, demonstrating the existence of visual persistence. It is consistent with the results that the normalized ΔI/I_0_ decreases gradually with the time. The visual persistence after a long illumination to mimic stare is significantly improved compared to a brief glance. The enhanced retention is evident from the image assessed immediately after the removal of signal for 5 s and also after a more extend period of 200 s. Moreover, the light pulse number from 2 to 30 is mimic to repeat watching. It demonstrates that effective memory can be preserved through repeated observations. Therefore, the as‐presented FeSFET with a simple transistor structure is capable of perceiving and memorizing images in response to light stimuli.

### 2.5 Neuromorphic Computing Simulation

**Figure 5:**
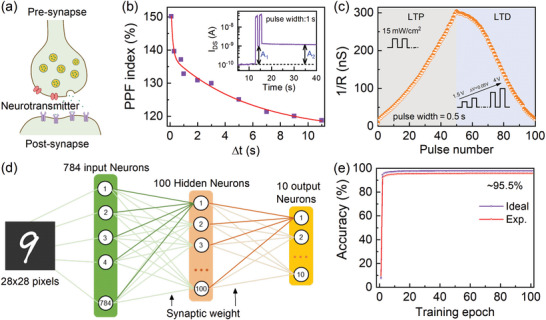
MNIST pattern recognition simulation of α‐In_2_Se_3_‐based FeSFETs. a) Schematic diagrams of a biological synapse. b) PPF index and the best‐fitted curve as a function of the paired pre‐synaptic spike interval in the P_up_ state. Inset: Current amplitudes (A_1_ and A_2_) caused by a pair of light pulses. c) Potentiation and depression with 100 consecutive pulses. d) Schematic of an ANN structure which is simulated with the standard back‐propagation algorithm. e) Recognition accuracy evolution as a function of training epochs for hand‐written MNIST digit images.

In neurobiology, two neighboring neurons are connected by a synapse, and signals are transmitted by chemicals. The presynaptic neuron releases neurotransmitters, which are then taken up by receptors on the postsynaptic neuron. Based on the characteristics of optical power‐dependent currents and multilevel current switching in α‐In_2_Se_3_ ferroelectric optoelectronic memories, the photonic synapse was naturally demonstrated by the emulations of synapse functions. **Figure** **5**a shows a human retinal synapse structure. Synaptic plasticity essentially has two basic features: short‐term plasticity (STP) and long‐term plasticity (LTP). Paired‐pulse facilitation (PPF) is an important factor in STP, which manifests itself as a heightened synaptic response elicited by two consecutive stimuli in biological synapses.^[^ ^51^ ^]^ In addition, PPF is associated with complex tasks performed by neurons that play an important role in decoding temporal information and audiovisual signals. As shown in Figure 5b, the PPF index of the α‐In_2_Se_3_ artificial synapse could be defined by the following formula:^[^ ^52^ ^]^ PPF = A_2_/A_1_ ×100%, where A_1_ and A_2_ are the current amplitudes after and before illumination, respectively. In Figure 5b, the PPF index decreases gradually from 150% to 118% with increasing Δt from 0.1 to 11 s. Additionally, parametric biexponential functions can model this behavior very well: The PPF index = a_0_ + a_1_ × exp (‐Δt/τ_1_) + a_2_ × exp (‐Δt/τ_2_)^[^ ^53^ ^]^ with two characteristic timescales τ_1_ = 127 ± 6 ms and τ_1_ = 5.7 ± 2.4 s, which are comparable to those measured in biological synapses.

The LTP and LTD demonstrate a continuous increase and decrease in the PSC under consecutive stimulations at a synapse, respectively. The LTP/LTD characteristic curves of α‐In_2_Se_3_‐based synapses for different light pulse widths have been investigated, as illustrated in Figure 5c. LTP is obtained by 50 light pulses at a fixed power density of 15 mW cm^−2^ in the P_up_ state. Under continuous pulsed light stimulation, the channel conductance gradually increases with increasing number of light pulses. On the other hand, LTD is measured by adjusting the amplitude of voltage pulses from 1.5 to 4 V with a step of 0.05 V. To demonstrate the application of our device to neuromorphic computation in artificial visual systems, a three‐layer ANN consisting of 784 input neurons, 300 hidden neurons, and 10 output neurons was constructed based on the LTP/LTD data (Figure 5d). With the help of the CrossSim simulator, the ANN was used to train and recognize handwritten digit images from the MNIST database. First, a 28 × 28 pixel handwritten digital image is classified by designing a single‐layer perceptron model with a backpropagation algorithm.^[^ ^54^ ^]^ Figure 5e shows that the recognition accuracy increases with the increase of training epoch. Note that a 91.79% recognition accuracy is achieved after the first training epoch. It finally reaches 95.55% after 100 training epochs, which is close to the accuracy of the ideal device (97.64%). Note that the recognition accuracy of our work is the highest among the α‐In_2_Se_3_ single‐material‐based FETs (cf. Table S1, Supporting Information).

## 3 Conclusion

In summary, a FeSFET has been successfully fabricated and has high electronic and optoelectronic performances due to the coexisting ferroelectric and semiconductor properties of 2H α‐In_2_Se_3_. The device exhibits distinct readout current levels, a extended retention time of over 5000 s and excellent cycle stability exceeding 2000 cycles. In a further step, the photoelectric coupling effects in 2H α‐In_2_Se_3_‐based FETs have been investigated systematically. The ΔI/I_0_ is 30 under 405 nm‐illumination in the P_down_ state, while it is enhanced to 390 in the P_up_ state. Moreover, the α‐In_2_Se_3_‐based FETs can be trained. It recognizes handwritten digit images from MNIST dataset with a high recognition accuracy of ≈95.5%. Therefore, the promising applications of the devices have been demonstrated in neuromorphic systems, particularly in image perception and visual persistence. These results suggest that ferroelectric nonvolatile memory holds significant promise for visual simulation and in‐memory sensing and computing.

## 4 Experimental Section

### Device Fabrication

The 2H α‐In_2_Se_3_ nanoflakes were obtained from the corresponding bulk crystals (Shanghai Onway Techology Co., Ltd) by mechanical exfoliation, and then transferred to 100 nm‐thick SiO_2_/p^++^‐Si substrates by dry transfer with polydimethylsiloxane (PDMS). The source and drain electrodes (20 nm/50 nm Cr/Au) were deposited by thermal evaporation using a copper mask.

### Device Characterizations

The morphology, thickness and piezoelectric force microscopy (PFM) of α‐In_2_Se_3_ nanoflakes were characterized by optical microscopy and atomic force microscopy (AFM, Dimension Icon, Bruker), respectively. The surface potentials of α‐In_2_Se_3_ were measured by kelvin probe force microscopy system (KPFM, Dimension Icon, Bruker). Raman spectra of α‐In_2_Se_3_ nanosheets and crystal were obtained by a confocal micro‐Raman spectrometer (Jobin‐Yvon LabRAM HR Evolution, Horiba) with a 532 nm‐laser. The electric and optoelectronic performances of α‐In_2_Se_3_‐based FeSFETs were investigated using a Keithley 4200‐SCS semiconductor parameter analyzer in a vacuum (10^−6^ Torr). All the electric and optoelectronic measurements in the probe station were carried out under dark conditions, and only exposed to the target light sources. In the photoelectric measurement process, commercial laser with illumination wavelengths of 405 nm was used (Thorlabs, Inc.). The laser spot area was ≈5 cm^2^. A laser diode controller (ITC4001, Thorlabs, Inc.) is used to produce laser pulses with tunable laser power density, pulse amplitude, pulse width, frequency, etc.^[^ ^55^ ^]^

## Conflict of Interest

The authors declare no conflict of interest.

## Author Contributions

C.Z. and Z.G. have contributed equally to this work. C.Z., Z.G., and Z.H. performed the preparation of 2D layered materials and the construction of FETs, C.Z., Z.G., and Z.C. conducted the experiments for device fabrication and electrical measurements, C.Z., L.S., L.Z., Y.L., and J.Z. performed the detailed analyses of the underlying mechanism, C.Z., J.Z.Z., and Z.G.H. designed the experiments and wrote the manuscript. J.Z.Z. supervised the research. All authors have given approval to the final version of the manuscript.

## Supporting information

Supporting Information

## Data Availability

The data that support the findings of this study are available from the corresponding author upon reasonable request.
