# Supplementary material for: Ferroelectric and Optoelectronic Coupling Effects in Layered Ferroelectric Semiconductor‐Based FETs for Visual Simulation
Source: Adv Sci (Weinh). 2025 Jan 22;12(11):2413808. doi: 10.1002/advs.202413808 (PMC11923961; doi:10.1002/advs.202413808)
Supplement: Supplementary file 1 — Supporting Information [file ADVS-12-2413808-s001.pdf]

ADVANCED SCIENCE
Open Access

Supporting Information

for *Adv. Sci.*, DOI 10.1002/advs.202413808

Ferroelectric and Optoelectronic Coupling Effects in Layered Ferroelectric Semiconductor-Based FETs for Visual Simulation

Can Zhao, Zhaotan Gao, Zian Hong, Hongzhi Guo, Zhili Cheng, Yawei Li, Liyan Shang, Liangqing Zhu, Jinzhong Zhang and Zhigao Hu**

Supplementary Information
Ferroelectric and Optoelectronic Coupling Effects in Layered Ferroelectric Semiconductor-based FETs for Visual Simulation

Can Zhao#,[1] Zhaotan Gao#,[1] Zian Hong,[1] Hongzhi Guo,[1] Zhili Cheng,[1] Yawei Li,[1] Liyan Shang,[1] Liangqing Zhu,[1] Jinzhong Zhang,[1,*] and Zhigao Hu[1,†]

[1] *Technical Center for Multifunctional Magneto-Optical Spectroscopy (Shanghai), Engineering Research Center of Nanophotonics & Advanced Instrument (Ministry of Education), Department of Physics, School of Physics and Electronic Science, East China Normal University, Shanghai 200241, China.*

(Dated: January 4, 2025)

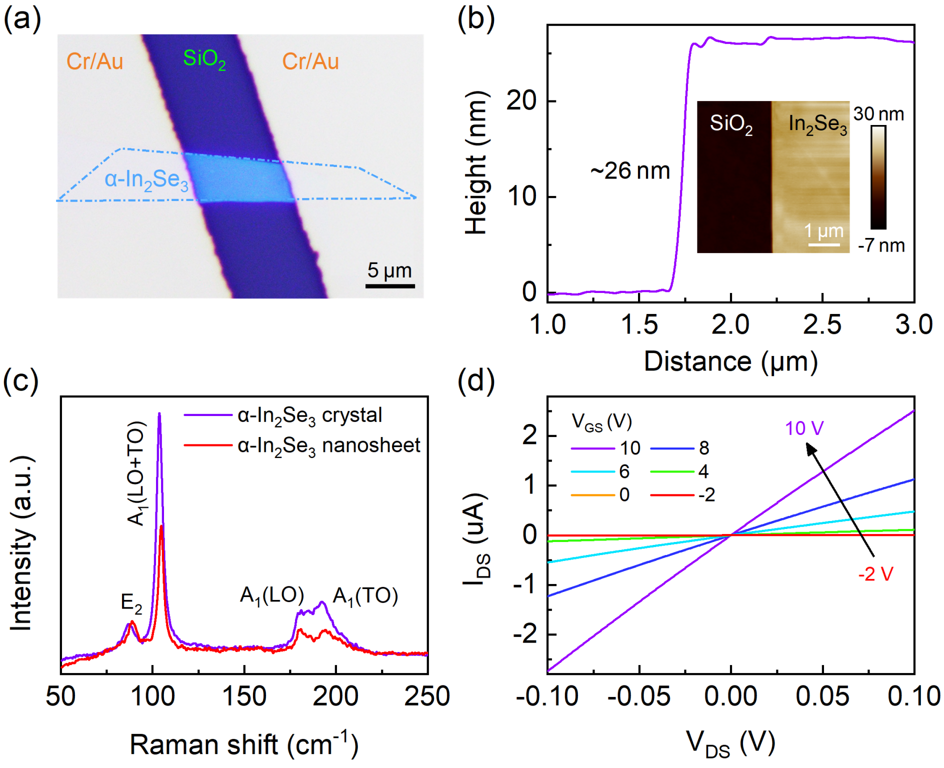

Figure S1: (a) An optical image of a α-In_2Se_3-based FeSFET. (b) An AFM image of a α-In_2Se_3 nanosheet on a SiO_2/p^{++}-Si substrate and the corresponding height profile. (c) Raman spectra of a 2H α-In_2Se_3 nanosheet and crystal. (d) I-V curves of a α-In_2Se_3-based FeSFET without illumination.

Figure S1a shows an optical image of a α-In_2Se_3-based FeSFET. The morphology of α-In_2Se_3 nanosheets as shown in Figure S1b has a clean and tidy surface, which is a prerequisite for the formation of high-quality devices. The corresponding height profile indicates that the thicknesses of α-In_2Se_3 nanosheet is about 26 nm. Figure S1c shows that Raman spectra of α-In_2Se_3 crystal and nanosheet exhibit four distinct peaks nearby 90, 106, 187 and 194 cm^{-1}, which are attributed to E_2, A_1(LO+TO), A_1(LO) and A_1(TO) first-order Raman-active modes of 2H α-In_2Se_3/SiO_2 with the space group $P6_3mc$, respectively. Note that the Raman peak nearby 90 cm^{-1} confirms the hexagonal structure (2H)[S1, S2]. The Raman spectra indicate that the nanosheets prepared by mechanical stripping method avoid the introduction of excessive defects compared to that of the corresponding crystals. The matching of the work function between α-In_2Se_3 and electrodes (20 nm/50 nm Cr/Au) could avoid the formation of Schottky barrier (cf. Figure S1d).

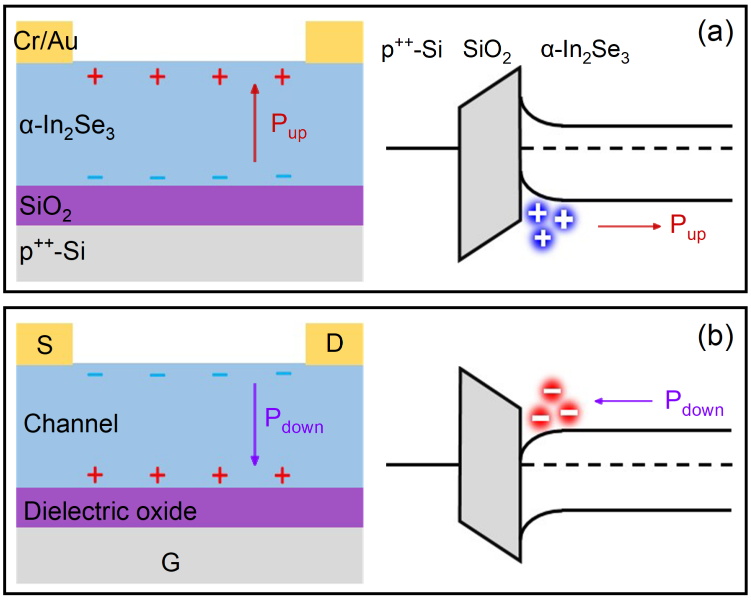

Figure S2: Illustrations of the (a) upward and (b) downward polarized states of 2H α-In_2Se_3 ferroelectric semiconductor channel and the corresponding energy band diagrams.

Figure S2 shows the distribution of polarization bound charge in the n-type FET channel and equilibrium band diagrams in the two polarization states (P_{up} and P_{down}). In the bottom-gate configuration as shown in Figure S2a, it induces an upward polarization in the α-In_2Se_3 layer when a $+V_{GS}$ is applied. Consequently, a sheet of negative-bonded charges at the interface between α-In_2Se_3 and SiO_2 is produced, resulting in the energy band bending upward[S1, S3]. Therefore, a depletion of the channel at the bottom occurs, which leads to a lower charge carrier density and channel current compared to the other two states. On the contrary, a -V_{GS} causes a downward polarization, leading to positive charges accumulating at the bottom of α-In_2Se_3 (Figure S2b). Therefore, the channel current in the P_{down} state is larger compared to those in P_{up} and fresh states. These variations in charge carrier density and channel current reflect the changes in polarization and associated band bending of the FeSFETs.

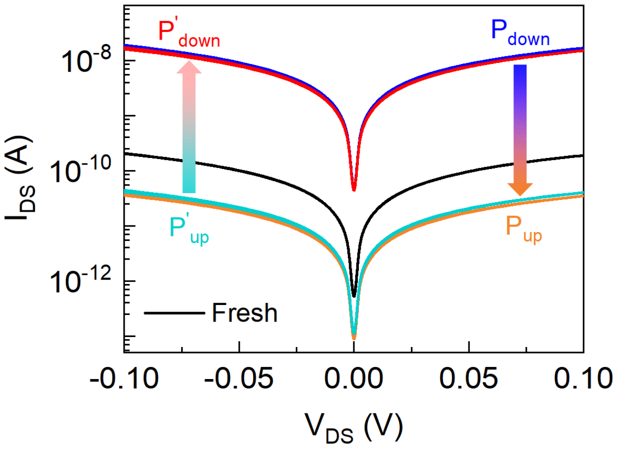

Figure S3: The output characteristic curves of the α-In_2Se_3-based ferroelectric transistor under the two polarization order ($P'_{up} \rightarrow P'_{down}$ or $P_{down} \rightarrow P_{up}$), $V_{GS-preset} = \pm 10\,V$.

In Figure S3, the output characteristic curves under the two polarization orders ($P'_{up} \rightarrow P'_{down}$ or $P_{down} \rightarrow P_{up}$) show that there is a similar current level. It indicates that the channel current in the α-In_2Se_3-based ferroelectric transistor is independent of the ferroelectric polarization order.

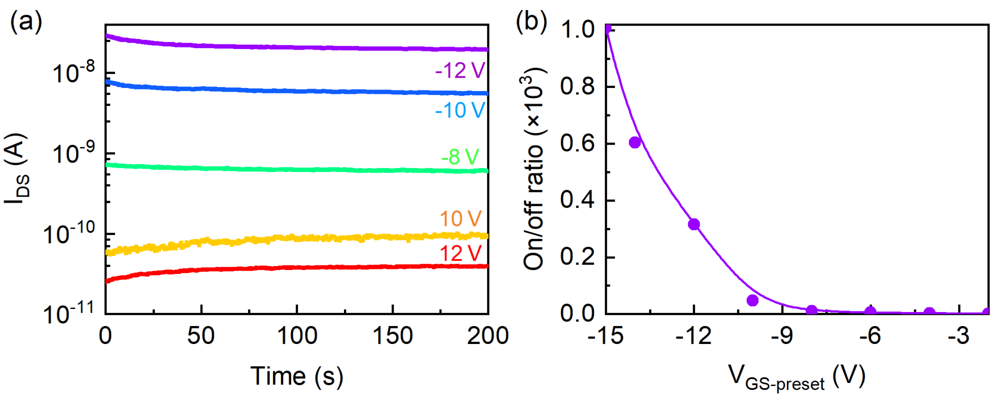

Figure S4: (a) Evolution of I_{DS} measured at $V_{DS} = 0.1\,V$ after applying a single gate voltage pulse with various amplitudes ($V_{GS-preset}$). (b) On/off current ratio as a function of $V_{GS-preset}$.

Figure S4a shows the retention characteristics at different amplitudes of gate voltage pulses ($V_{GS-preset}$) with the pulse width of 700 ms. Due to the remnant polarization of ferroelectric α-In_2Se_3, the channel current is suppressed and reduced to below 10^{-10} A even after the positive $V_{GS} = +10\,V$. Meanwhile, the I_{DS} cannot recover to its initial value in the measurement time range (200 s) after a negative V_{GS} spike. In addition, increasing the amplitude of V_{GS} pulses will result in a large on/off current ratio of I_{DS} measured in a P_{down} state (cf. Figure S4b).

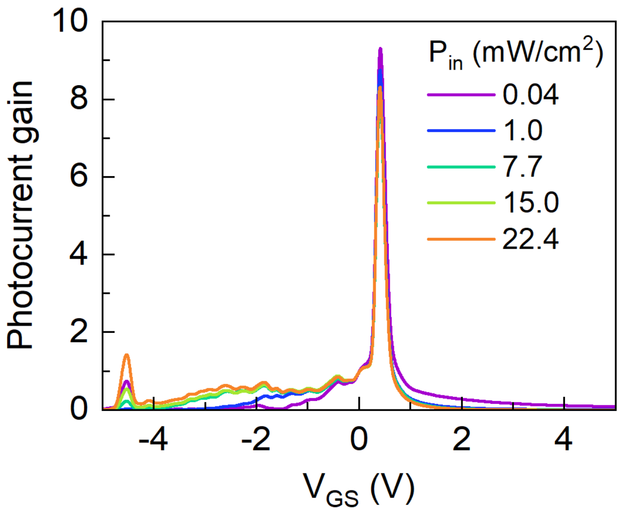

Figure S5: Photocurrent gain as a function of gate voltage at $P_{in} = 0.04 \sim 22.4\,mW/cm^2$.

The photocurrent gain (G) shown in Figure S5 was derived by the following formula[S4]: $G(V) = (I_{photo(V)} - I_{dark(V)})/(I_{photo(V=0)} - I_{dark(V=0)})$. It suggests that the photocurrent gain at different gate voltages is different at the same laser power density. As the laser power density increases, the photocurrent gain increases at negative V_{GS}, while decreases at positive V_{GS}. Therefore, the α-In_2Se_3-based FeSFET has different photocurrent gain under positive and negative gate voltages due to the difference of α-In_2Se_3 out-of-plane polarization.

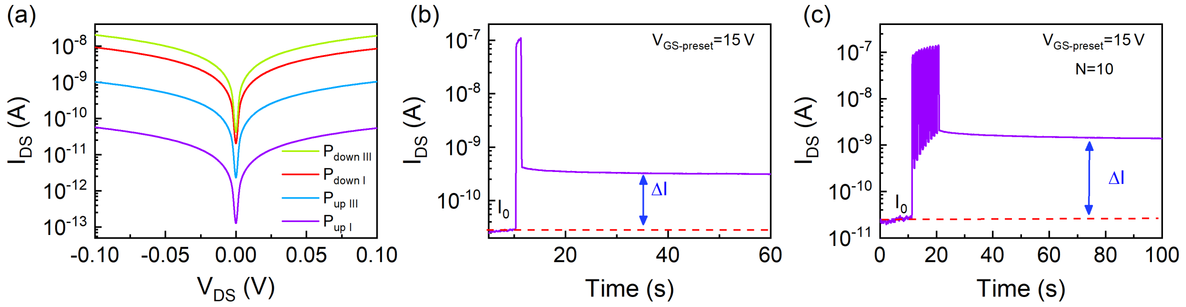

Figure S6: (a) Output characteristic curves (I) before and (III) after illumination in the P_{down} and P_{up} states. The I_{DS} at $V_{DS} = 0.1$ V before, during, and after (b) a single ($\lambda = 405$ nm, $P_{in} = 15$ mW/cm^2, pulse width: 3 s) and (c) multiple light pulses (pulse number: 10, pulse width: 0.5 s) in a P_{up} state.

Figure S6a shows the I_{DS}-V_{DS} curves of a FeSFET under two conditions: (I) before and (III) after illumination at $P_{in} = 15$ mW/cm^2 in two ferroelectric polarization states (P_{down} and P_{up}). It indicates that the I_{DS} after illumination is larger than that before illumination in both polarized states. In a further step, Figure S6b and S6c show the I_{DS} response at $V_{DS} = 0.1$ V before, during, and after a single light pulse. Here the $\Delta I/I_0$ is used to assess the retention capability after applying light pulses in various polarization states. Note that I_0 is the I_{DS} before illumination and ΔI is the difference of I_{DS} before and after illumination.

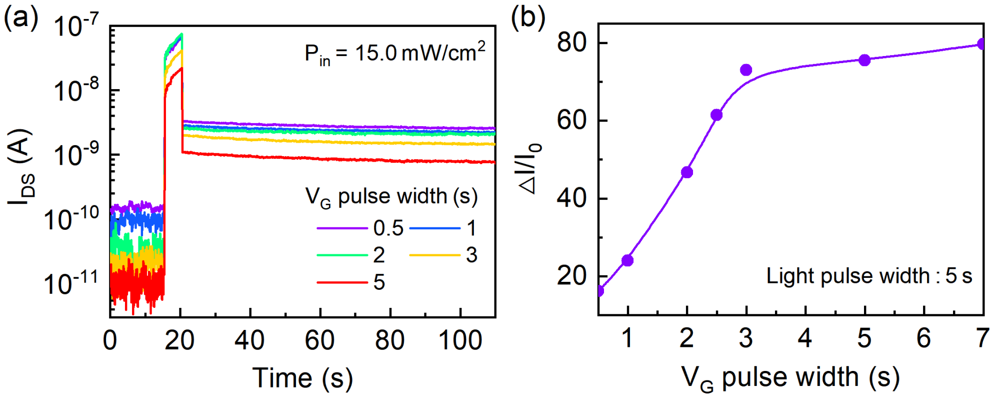

Figure S7: (a) Photocurrent curves and (b) charge retention ability of a α-In_2Se_3-based FeSFET at various gate voltage pulse widths at $P_{in} = 15.0\,mW/cm^2$.

The influence of polarization voltage pulse and extraction time on the photocurrent of α-In_2Se_3-based FesFETs has been investigated by changing the pre-gate voltage pulse width, as shown in Figure S7a. It shows that the device has good retention ability under different pre-gate voltage pulse widths. Figure S7b suggests that the charge retention ability of the α-In_2Se_3-based FeSFET is stable after applying the pre-gate voltage pulse. In order to exclude the influence of data extraction, the data points evaluating charge retention ability were taken from the first data point after illumination.

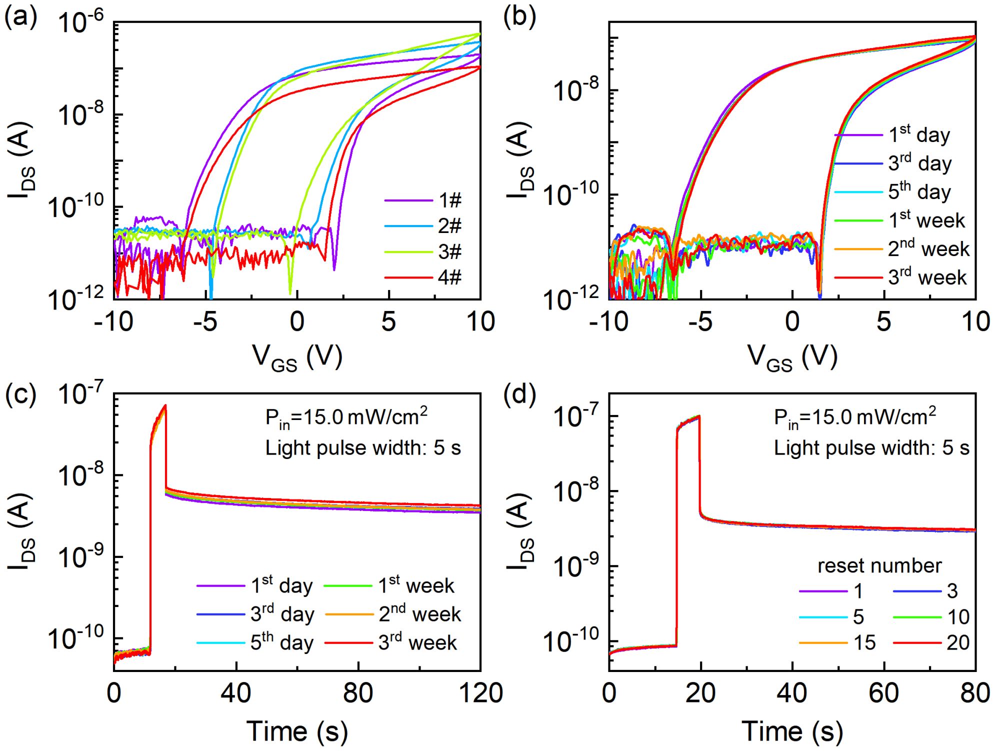

Figure S8: (a) Transfer characteristic curves of different α-In_2Se_3-based FesFETs. (b) Transfer characteristic curves and (c) photocurrent curves in a period of time ($P_{in} = 15\,mW/cm^2$, $V_{GS-preset} = 10\,V$). (d) I_{DS} as a function of time under multiple resets.

In Figure S8a, the transfer characteristic curves of different α-In_2Se_3-based ferroelectric FETs indicates that this kind of device has a good repeatability. Moreover, it shows a considerable hysteretic window. Figure S8b shows the cyclic stability of the α-In_2Se_3-based FeSFET for three weeks. The hysteretic window is almost the same over three weeks, which indicates that the device has a stable performance over a period of time. In Figure S8c, the device photocurrent remains basically the same for three weeks, and the comparison results with the initial state show that the photocurrent changed in the range of 4%. In addition, the band and retention levels are also in the 21% range. It means the FeSFET has excellent cyclic and environmental stabilities. Figure S8d shows that the channel current is almost the same and still maintains excellent charge retention ability after 20-cycle tests. It indicates that the α-In_2Se_3-based FeSFET has good cyclic characteristics.

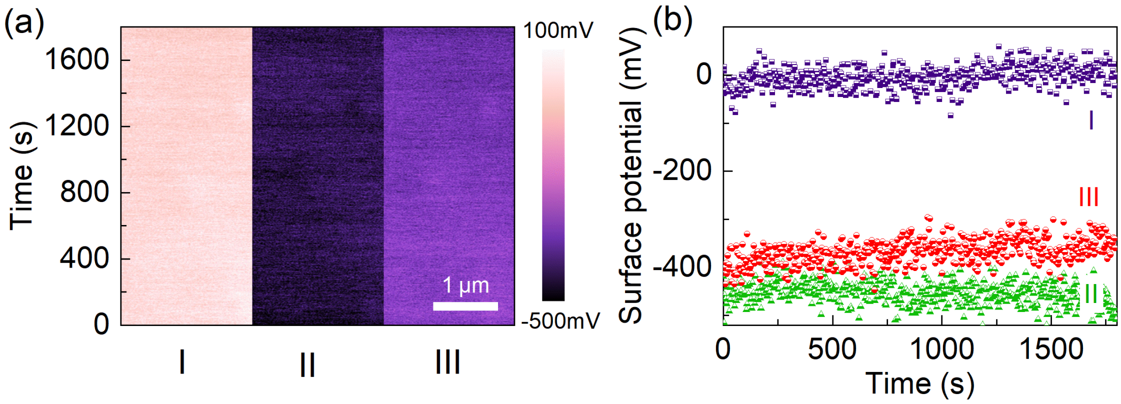

Figure S9: Time-dependent surface potentials of α-In_2Se_3 under the three illumination conditions: (I) before illumination, (II) during illumination, and (III) after illumination.

In Figure S9a, the time-dependence of surface potentials under the three illumination conditions (I, II, and III) shows a uniform change for 30 minutes. It indicates the surface potentials have not an obvious decline under the same illumination condition. Moreover, the values have an obvious difference in difference illumination conditions, as shown in Figure S9b. Therefore, the surface potential is mainly affected by the illumination conditions.

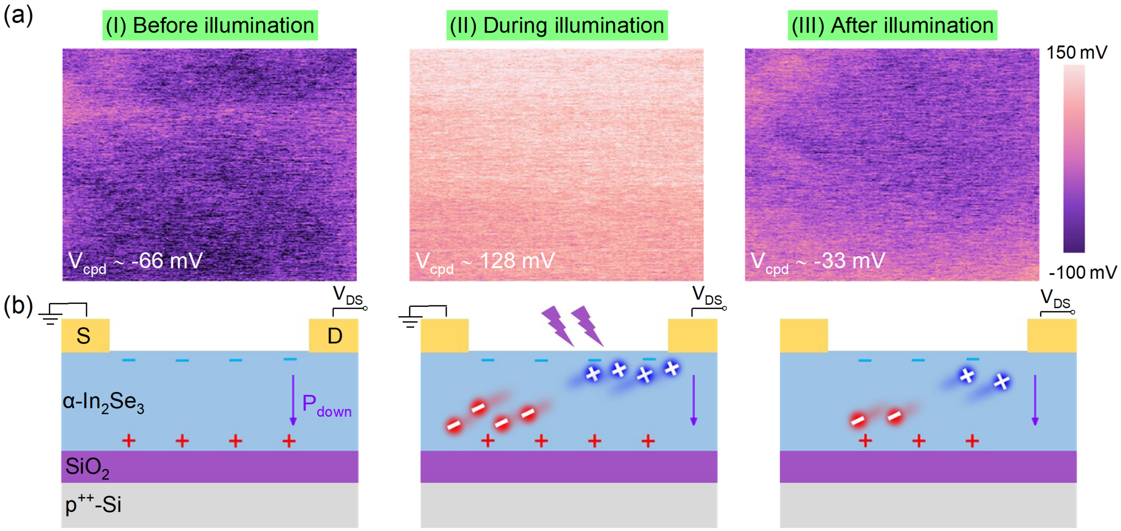

Figure S10: (a) KPFM surface potential images and (b) the corresponding distributions of ferroelectric polarization- and photon-induced carriers under the three light conditions (I) before, (II) during, and (III) after illumination in a P_{down} state.

Figures S10a shows KPFM of α-In_2Se_3 in a P_{down} state under the three illumination conditions (I, II, and III) to reveal the carrier modulation and optoelectronic responses. The average V_{CPD} varies from -66 mV (I) to 128 mV (II), and then to -33 mV (III). Note that V_{CPD} in the III condition remains higher than the initial level under the I condition. The distribution of polarization charge and photon-induced carriers in the channel under the three light conditions is shown in Figure S10b. The accumulation of negative bound charges on the channel (α-In_2Se_3) surface results in a relatively negative surface potential before illumination. And the built-in electric field (E_{in}) generated by ferroelectric polarization significantly influences photon-induced carriers. The semiconductor/dielectric interface (α-In_2Se_3/SiO_2) will accumulate photon-generated electrons, while positive bound charges on the top surface of α-In_2Se_3 result in a positive shift.

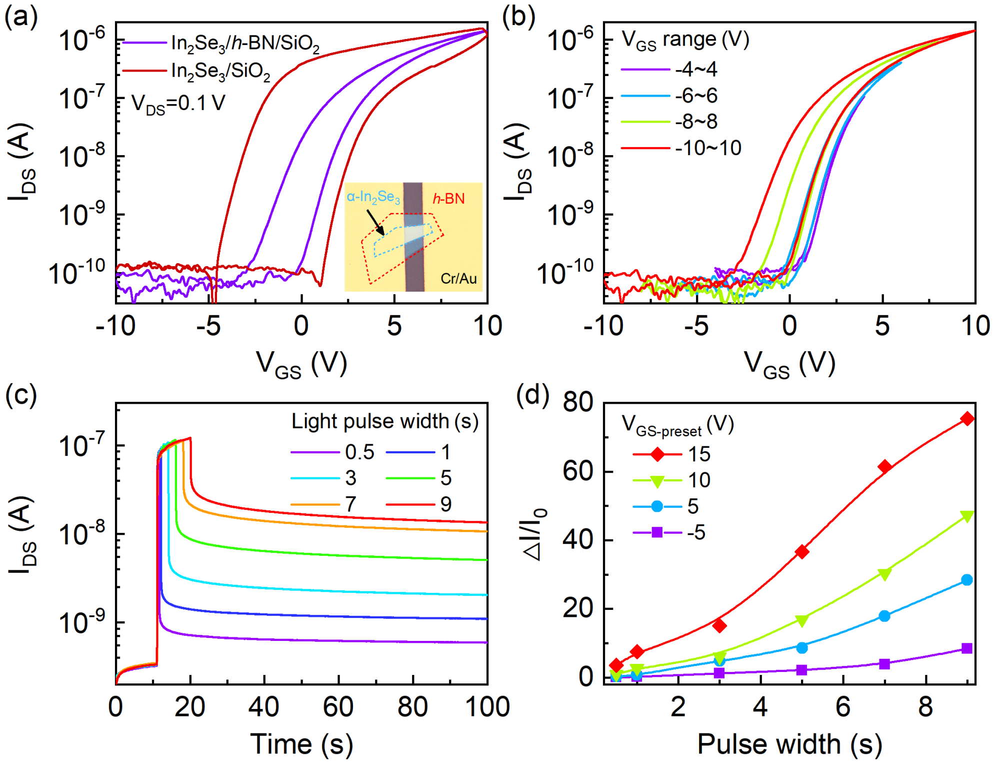

Figure S11: (a) Transfer characteristics curves of α-In_2Se_3/h-BN/SiO_2- and α-In_2Se_3/SiO_2-based FeSFETs at $V_{DS} = 0.1$ V. (b) I_{DS} of a α-In_2Se_3/h-BN/SiO_2-based FeSFET by applying various V_{GS} ranges. (c) Time-dependent I_{DS} by applying a single pulsed light with different light pulse widths ($P_{in} = 15.0$ mW/cm^2) after a V_{GS} pulse ($V_{GS-preset} = 10$ V, pulse width: 3 s) and (d) the corresponding $\Delta I/I_0$ as a function of light pulse width.

In order to eliminate the interface effect between α-In_2Se_3 and SiO_2, a In_2Se_3/h-BN/SiO_2 device structure with a 20 nm-thick layered h-BN is fabricated, as shown in the inset of Figure S11a. In Figure S11a, the transfer characteristic curves show that the FeSFET with h-BN dielectric layer has a smaller hysteretic window. It indicates that the presence of h-BN dielectric layer could effectively reduce the influence of interface defects between In_2Se_3 and SiO_2. Figure S11b shows the I_{DS} of a In_2Se_3/h-BN-based FeSFET in different gate voltage ranges. It suggests that the FET still maintains a hysteresis window. Figure S11c shows that the In_2Se_3/h-BN-based FeSFET exhibits non-volatile conductivity at different pulse widths. With the increase of light pulse width, it has a higher current level after illumination, which means it has a good charge retention capability. Figure S11d shows the charge retention capacity of a In_2Se_3/h-BN FeSFET at different laser pulse widths and $V_{GS-preset}$. At the

same laser pulse width, the charge retention ability increases gradually with the $V_{GS-preset}$. It means that the charge retention ability is closely related to the polarization degree of In_2Se_3. At the same $V_{GS-preset}$, the charge retention ability increases with the pulse width, indicating that the charge retention capacity is positively correlated with the amount of photogenerated charge. Therefore, the charge retention ability of In_2Se_3/*h*-BN-based FeSFETs is the result of the coupling between ferroelectric polarization field and photogenerated charge. The 2D *h*-BN layer does not affect the coupling between ferroelectric polarization field and the photogenerated charge in In_2Se_3/*h*-BN-based FeSFETs. Note that the 2D *h*-BN interlayer between In_2Se_3 and SiO_2 could avoid the charge capture resulting from the interface defect between In_2Se_3 and SiO_2. In summary, the non-volatile photoconductivity in In_2Se_3-based FeSFETs is mainly caused by ferroelectric polarization and photoelectrochemical charge instead of the charge trapping by the interface defects between In_2Se_3 and SiO_2.

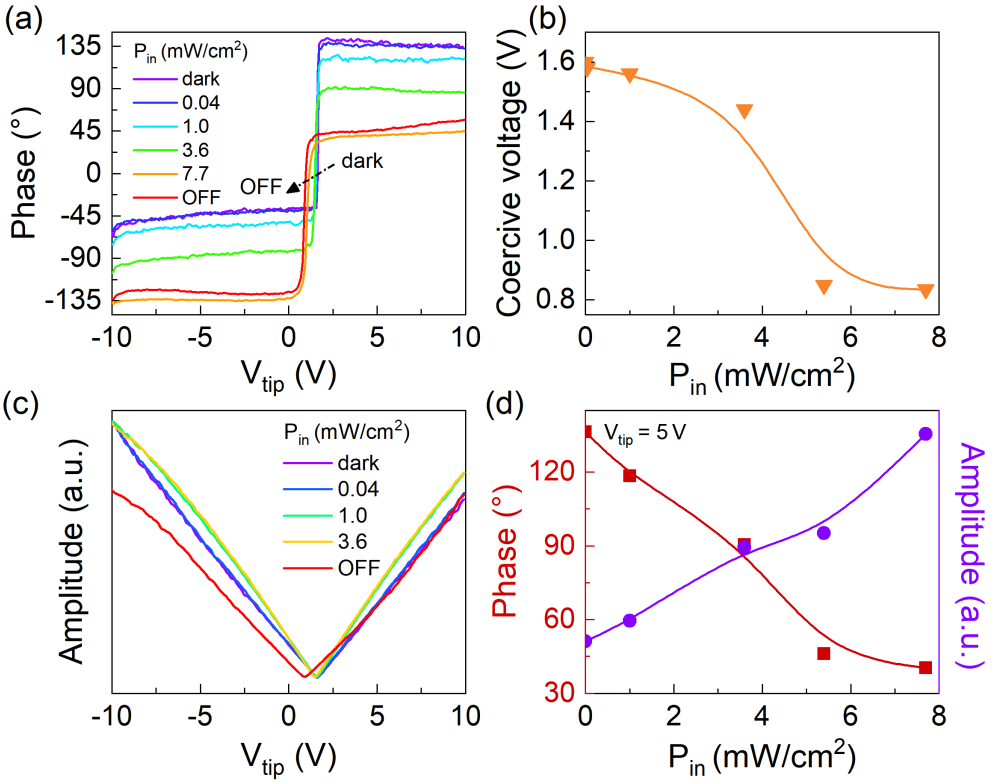

Figure S12: (a) PFM phase curves at various laser power densities. (b) Coercive voltage as a function of laser power density. (c) PFM amplitude curves at various laser power densities. (d) Piezoelectric phase and piezoelectric amplitude at $V_{tip} = 5$ V as a function of laser power density.

Figure S12a shows PFM phase curves at various laser power densities. It indicates that complete polarization switching occurs by the near 180° contrast phase curves. Since semiconducting ferroelectric α-In_2Se_3 has an excellent photoelectric effect, illumination enables a drastic increase of the photogenerated electrons and holes. Then the separation and transfer of photogenerated carriers shields a part of the built-in electric field. Therefore, the coercive voltage is reduced, as shown in Figure S12b. The photogenerated carriers would not disappear immediately after illumination. The carriers persist inside the channel for a certain time due to the polarization state. The PFM amplitude shown in Figure S12c increases with increasing the laser power density. Figure S12d shows the corresponding piezoelectric phase and amplitude. The direction of the built-in electric field generated by the photogenerated charges is the same as that of polarization field in P_{up} and P_{down} states, which enhances the amplitude of polarization field.

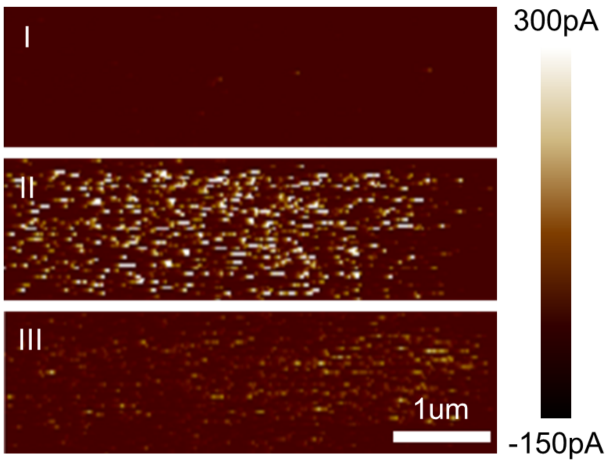

Figure S13: Photocurrent mapping of a α-In_2Se_3-based FET (I) before, (II) during, and (III) after illumination in the P_{up} state.

Figure S13 shows the photocurrent mapping (I) before, (II) during, and (III) after illumination in the P_{up} state. For the case of I (before illumination), there is no obvious current signal in the P_{up} state, showing a more uniform low current distribution or no current region. The ferroelectric semiconductor is in a thermal equilibrium and the internal carriers without photogenerated carriers are in a stable state. During illumination (II), the photocurrent mapping appears to have an uneven distribution of photocurrents, with higher photocurrent densities in some areas and lower ones in others. It means the illumination on the α-In_2Se_3 device enables a drastic increase of the photogenerated electrons and holes. Due to the ferroelectric properties of α-In_2Se_3, there is a built-in electric field inside, which causes the photogenerated electrons and holes to move in the opposite direction, resulting in a photocurrent. After illumination (III), the photocurrent does not disappear since the photogenerated electrons and holes would not re-combinate immediately due to the ferroelectric polarization.

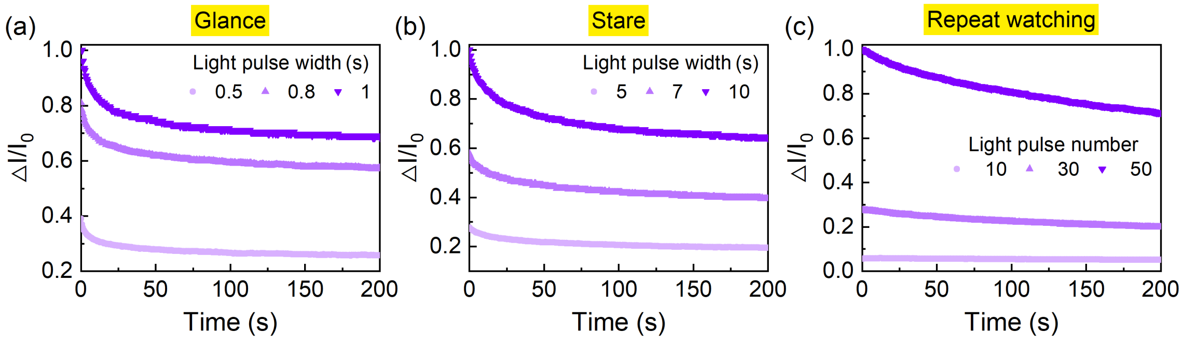

Figure S14: Normalized $\Delta I/I_0$ after a single laser pulse with the pulse width (a) below and (b) above 1 s, and (c) pulse numbers from 10 of 50 (pulse width: 0.5 s).

In Figure S14, the normalized $\Delta I/I_0$ increase as the pulse width and number increase and decays slowly after illumination. When the device presents a brief impression of the letter "U", similar to a short period of illumination (< 1 s) that simulates glancing light (glance), it shows a recognizable change in current. $\Delta I/I_0$ decreases gradually with the time. Even after the visual input signal for 200 s, the "U" shape is retained, demonstrating the existence of visual persistence phenomenon. When observed with much attention (stare), such as during longer light exposure (pulse width : > 1 s), the memory retention is significantly improved compared to a brief glance. Additionally, $\Delta I/I_0$ after the illumination can be regulated by the number of light pulses and gradually increases with the number of stimuli. As the optical pulse number increases from 10 to 50, the clarity of the letter of "U" is enhanced. It demonstrates that effective persistence can be preserved through repeat watching.

Table S1: The comparison of neuromorphic characteristics.

Device structure	Program voltage (V)	On/Off ratio	R (A/W)	Recognition accuracy (%)	Refs.
In_2Se_3/SiO_2	40	10^2	–	87	[S5]
In_2Se_3/HfO_2	5	10^6	2855	92.6	[S6]
In_2Se_3/Al_2O_3	10	10^5	–	91.9	[S7]
In_2Se_3/HfO_2	5	10^5	1.2×10^5	94	[S8]
In_2Se_3/SiO_2	10	10^5	20	95.5	Our work

The values of on-off ratio and recognition accuracy of the α-In_2Se_3-based FETs have been summarized in Table S1. Note that the recognition accuracy of our work is the highest among the α-In_2Se_3 single-material-based FETs.

* Electronic address: jzzhang@ee.ecnu.edu.cn

† Electronic address: zghu@ee.ecnu.edu.cn

[S1] Y. Chen, D. Li, H. Ren, Y. Tang, K. Liang, Y. Wang, F. Li, C. Song, J. Guan, Z. Chen, X. Lu, G. Xu, W. Li, S. Liu, and B. Zhu, Small **18**, 2203611 (2022).

[S2] M. Küpers, P. M. Konze, A. Meledin, J. Mayer, U. Englert, M. Wuttig, and R. Dronskowski, Inorg. Chem. **57**, 11775 (2018).

[S3] L. Wang, X. Wang, Y. Zhang, R. Li, T. Ma, K. Leng, Z. Chen, I. Abdelwahab, and K. P. Loh, Adv. Funct. Mater. **30**, 2004609 (2020).

[S4] X. Li, J. Chen, F. Yu, X. Chen, W. Lu, and G. Li, Nano Lett. **24**, 13255 (2024).

[S5] J. Shin, J. Jang, C. H. Choi, J. Kim, L. Eddy, P. Scotland, L. W. Martin, Y. Han, and J. M. Tour, Adv. Electron. Mater. **10**, 2400603 (2024).

[S6] G. Wu, L. Xiang, W. Wang, C. Yao, Z. Yan, C. Zhang, J. Wu, Y. Liu, B. Zheng, H. Liu, C. Hu, X. Sun, C. Zhu, Y. Wang, X. Xiong, Y. Wu, L. Gao, D. Li, A. Pan, and S. Li, Sci. Bull. **69**, 473 (2024).

[S7] L. Wang, X. Wang, Y. Zhang, R. Li, T. Ma, K. Leng, Z. Chen, I. Abdelwahab, and K. P. Loh, Adv. Funct. Mater. **30**, 2004609 (2020).

[S8] N. T. Duong, Y. Shi, S. Li, Y.-C. Chien, H. Xiang, H. Zheng, P. Li, L. Li, Y. Wu, and K.-W. Ang, Adv. Sci. **11**, 2303447 (2024).
